# Bioconversion of Liquid and Solid Lipid Waste by *Yarrowia lipolytica* Yeast: A Study of Extracellular Lipase Biosynthesis and Microbial Lipid Production

**DOI:** 10.3390/molecules30040959

**Published:** 2025-02-19

**Authors:** Katarzyna Wierzchowska, Karolina Szulc, Bartłomiej Zieniuk, Agata Fabiszewska

**Affiliations:** 1 Department of Chemistry, Institute of Food Sciences, Warsaw University of Life Sciences-SGGW, 159c Nowoursynowska Street, 02-776 Warsaw, Poland; katarzyna_wierzchowska1@sggw.edu.pl (K.W.); bartlomiej_zieniuk@sggw.edu.pl (B.Z.); 2 Department of Food Engineering and Process Management, Institute of Food Sciences, Warsaw University of Life Sciences-SGGW, 159c Nowoursynowska Street, 02-776 Warsaw, Poland; karolina_szulc1@sggw.edu.pl

**Keywords:** *Yarrowia lipolytica*, lipid wastes, microbial lipids, lipases, inoculum, emulsification activity, particle size distribution

## Abstract

This study investigated the capabilities of *Yarrowia lipolytica* strains to grow in media with different hydrophobic wastes from the meat industry. The yeast growth, cellular lipid accumulation, production of lipases, and degree of utilization of liquid and solid lipid wastes were studied in shaken cultures in media with organic and inorganic nitrogen sources. The effects of the type of waste, initial concentration of carbon source, *Yarrowia* strain, and inoculum size were investigated in two experimental sets using the Latin Square 5 × 5 design method. Post-frying rapeseed oil from chicken frying was selected as the carbon source to promote biomass growth. In contrast, the solid lipid fraction from meat broths promoted efficient lipid accumulation and yeast lipolytic activity. An initial concentration of the carbon source at 8% *m*/*v* stimulated efficient lipid biosynthesis and lipase production, while 2.5% *v*/*v* inoculum provided optimal conditions for the growth and utilization of hydrophobic substrates. No significant differences were observed in the particle dispersion of the liquid and solid wastes in the culture media (*span* = 2.51–3.23). The maximum emulsification index (62%) was observed in the culture of the *Y. lipolytica* KKP 323 strain in the medium with post-frying rapeseed oil from chicken frying, which was correlated with biosurfactant synthesis. It was concluded that the type of waste, its structure, and its composition affected various physiological yeast responses.

## 1. Introduction

The total amount of food waste is difficult to estimate, even though it represents the main part of all municipal waste [1]. However, the United Nations Environment Programme—Food Waste Index Report 2021 estimated the value of food waste generated in 2019 to be about 931 million tons, of which 61% came from households and 26% from food services [2]. Fat waste is a distinct category within the field of waste management. Due to the structural and physico-chemical properties of fats, unique difficulties must be addressed when planning their disposal. Animal fats solidify at low temperatures and melt at high temperatures. Vegetable oils tend to have a higher viscosity. At the same time, solid animal fats may occur in a semi-solid or paste state at room temperature, making managing this waste more challenging [3,4].

Various waste materials have been utilized as cost-effective alternative substrates for the microbial production of many metabolites that can find industrial applications [5]. This approach provides a dual benefit: waste disposal and synthesizing value-added products. Much scientific research on the biotechnological management of various types of wastes, including fatty ones, is focused on the use of the oleaginous species *Y. lipolytica*, mainly because of their extraordinary ability to grow in media containing complex carbon sources, both hydrophilic and hydrophobic, and its high tolerance to a wide range of salt concentrations and pH values [6]. *Y. lipolytica* is also known for the extracellular lipolytic activity that enables the hydrolysis of triglycerides to free fatty acids and glycerol at the oil–water interface in the culture media [7]. Due to the ability to catalyze specific biotransformation in various reaction mixtures, lipases are used in industries ranging from pharmaceuticals to food and chemicals [8]. Lipases have been widely used as biocatalysts in multiple reactions, such as esterification, transesterification, interesterification, thiotransesterification, alcoholysis, acidolysis, aminolysis, and oxymolysis [9,10]. Fungi are recognized as one of the most effective sources of lipase among microorganisms. Because of the ability of fungal lipases to remain active under a wide range of pHs, extreme temperatures, and organic solvents, enzymes are capable of catalyzing a variety of chemical reactions [10,11,12,13,14].

*Y. lipolytica* yeast may also produce biosurfactants to reduce the size of hydrophobic substrate droplets, thereby increasing the contact surface area with the yeast cell. Biosurfactants are biodegradable, amphiphilic molecules with surface-active properties commonly used in the textile industry, bioremediation, agriculture, or food and beverage industries [7,15,16]. Free fatty acids, either available in the substrate or derived from the hydrolysis of other hydrophobic substrates, are actively transported into the cell, where they can undergo various enzymatic modifications, including the β-oxidation process [17].

Fatty wastes may become substrates in the microbial synthesis of new products. Microbial lipids have recently gained considerable attention to meet the demand for lipids as feedstock for biodiesel, dietary supplements, or food enrichment ingredients, mainly because of the fatty acid composition of microbial lipids, which are similar to vegetable oils, both in terms of the chain length and degree of saturation [18]. It was shown that biodiesel derived from the microbial lipids of *Y. lipolytica* is of high quality and complies with international biodiesel standards [19].

Biotechnological processes based on *Y. lipolytica* yeast have been classified as Generally Recognized as Safe (GRAS) by the Food and Drug Administration (FDA). The European Food Safety Authority (EFSA) has approved its biomass, cultured in waste media, as a novel food for use in dietary supplements for those over 3 years old, with daily limits of 3 g for children (3–9 years) and 6 g for older individuals. Regulation 2024/2044 outlines its permissible use in various food products, including meal replacements, medical foods, dairy, bread, or soup [20].

Taking into account literature reports and emerging regulations on *Y. lipolytica* yeast, it can be stated that this issue raises the interest of both researchers and industry. Thus, it is worth investigating not only the effects of substrates and culture parameters on the phenotypic characteristics of the yeast but also the ability of the cells to degrade hydrophobic industrial wastes of varying consistencies and structures, which are difficult to dispose of. For this reason, this work investigated the capabilities of *Y. lipolytica* strains to grow in media with different hydrophobic wastes from the food industry to evaluate their potential as producers of microbial lipids and lipases. The selection of liquid and solid lipid wastes was made to choose the one that guarantees the most efficient biosynthesis of investigated microbial products. Differences in the phenotypic traits of yeast strains, namely, SCO synthesis efficiency, biomass yield, and lipolytic activity, were compared. Additionally, the emulsification activity was measured to investigate the waste substrates’ degradation and the yeast’s ability to modulate the degree of emulsification of complex two-phase media. Furthermore, granulometric analyses of the fatty droplet size distribution in the medium were conducted to show the degree of substrate decomposition.

## 2. Results and Discussion

### 2.1. Selection of Lipid Waste Carbon Sources Easily Utilized by Y. lipolytica Yeast Cells

It has been known that hydrophobic waste may be effectively utilized by *Y. lipolytica* yeast cells. Waste cooking oils (WCOs) are fats and oils discarded after food processing, of which 1 million tonnes are produced worldwide [21]. Managing this type of waste is challenging due to the storage difficulties and the risk of ecosystem contamination. WCOs may be diversified in composition, such as the fatty acid profile or other bioactive compounds. It depends on the type of fat used in the frying process and the raw material processed [5]. In the first experiment, lipid wastes from the agri-food industry were provided to choose the best one to stimulate the growth and cellular lipid accumulation of *Y. lipolytica* yeast. All the wastes served as a source of carbon for oleaginous yeast. The cultures were conducted in 25 variants, and the type of waste, their concentrations, and *Yarrowia* strain were varied according to the experimental plan, asdescribed in the Materials and Methods section.

**Figure 1:**
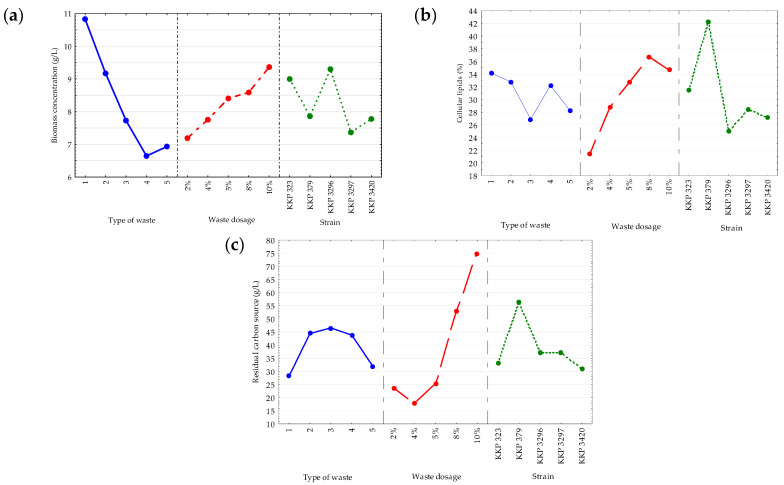
Main effects plot for the (**a**) biomass concentration (g/L), (**b**) cellular lipid content (%), and (**c**) residual carbon sources content in the medium (g/L) when the cultures in the media with peptone and yeast extract varied in the types of waste, the waste concentrations, and *Y. lipolytica* strains. 1—post-frying rapeseed oil from chicken frying, 2—waste lard from pork frying, 3—waste fat from pork head cooking, 4—solid lipid fraction from meat broths, and 5—post-frying rapeseed oil from chicken frying + waste lard (50:50). All of the culture variants were incubated at 28 °C for 96 h.

A significant correlation between the biomass growth and the type of waste carbon source in the culture medium was noted (*p-*value = 0.002) (Figure 1a). Comparing the results for cultures with liquid and solid lipid substrates, the yeast grew most efficiently in media with post-frying rapeseed oil from chicken frying (10.76 g/L). The lowest biomass concentration was observed in the medium with a solid lipid fraction from meat broths (6.64 g/L). High biomass yield values (14.40–26.67 g/L) were noted in media that contained oil industry residues and various oils (e.g., rapeseed, sesame, borage). Such final biomass concentrations were reflected in a large amount of microbial lipids that were possible to obtain from the volume of the medium (11–16 g/L), despite the fact that the lipid content in the dry cell mass (32–62%) was at a level comparable with this and other studies, also cited in the following section [22].

Considering the effects of the initial waste dosages and strain types on the biomass concentration, the observed correlations were not statistically significant (*p*-value > 0.05) under the assumed experimental conditions. However, it can be said that a higher addition of hydrophobic carbon sources (8–10%) was correlated with better biomass growth. The highest biomass concentrations were found in the cultures of KKP 323 and KKP 3296 strains (Figure 1a).

In the experiment, the additional impact of *Yarrowia* strains and initial waste dosage influenced the efficiency of the microbial lipid biosynthesis (for the yeast strain, *p*-value = 0.002; for the level of the lipid carbon source in media, *p*-value = 0.007) (Figure 1b). The highest cellular lipid accumulation efficiency was observed when the yeast was cultured in an 8% carbon source medium. The microbial lipid biosynthesis efficiency was above 20% for all the culture variants. The best producer of microbial lipids under the experimental conditions used was the *Y. lipolytica* strain KKP 379.

In an experiment designed using the Taguchi L9 orthogonal array, Lopes et al. [23] cultured *Y. lipolytica* W29 in media supplemented with pork lard (2–8%). The accumulation efficiency ranged from 26.4 to 87.9% with a biomass concentration of 3.7–8.6 g/L. In contrast to the current study, the initial content of carbon sources had no significant effect on microbial lipid production in the medium with yeast extract as a nitrogen source. Similarly, the pH and the addition of arabic gum were not significantly related to the accumulation efficiency. In our study, the pH was not adjusted, and the initial addition of peptone, as well as the yeast extract, was constant. The initial content of the lipid carbon source was modified, so the C/N ratio of the experimental media differed between experiments. The results obtained stand in opposition to the results presented by Lopes et al. [23], Donot et al. [24], and Papanikolaou and Aggelis [25], where the C/N ratio had no effect on the process of cellular lipid production in media with hydrophobic carbon sources. The effects of nitrogen and phosphorus limitation has been described in earlier reports [26].

In order to optimize the culture process of the *Y. lipolytica* strain CICC1778 in media with mutton fat, Xiong et al. [27] evaluated the effect of waste concentration as a carbon source (1, 3, 5%) and (NH_4_)_2_C_4_H_4_O_6_ as a nitrogen source on growth and lipid production. Initial concentrations of mutton fat (3%) and nitrogen source (3 g/L) were chosen as optimal for the growth (14.11 g/L) and cell lipid content (33.1%, *w*/*w*). The authors noted that at a higher concentration of lipid substrate (50 g/L), a large portion of the carbon source remained unused [27]. In the current study, the residual carbon sources in the medium after the yeast culture were also correlated with the waste dose (*p*-value = 0.0000) (Figure 1c). Post-frying rapeseed oil from chicken frying proved to be the substrate the cells best assimilated with. This was evidenced by the lowest carbon source level in the media after 96 h of culture. When higher waste dosages were used, more waste substrates remained unused in the medium. The strain also proved to be a factor that significantly affected the level of waste carbon source in the medium. The highest proportion of residual carbon sources was observed in the media in which strain KKP 379 was cultured.

### 2.2. Selection of Yeast Strain and Inoculum Size

In the second experimental set of cultures, a mineral medium with a reduced nitrogen content was the base for all media variants that stimulated efficient ex novo lipid accumulation by yeast cells. The effects of the carbon source, inoculum size, and oleaginous yeast strain on the biomass growth and cellular lipids biosynthetic efficiency were evaluated.

**Figure 2:**
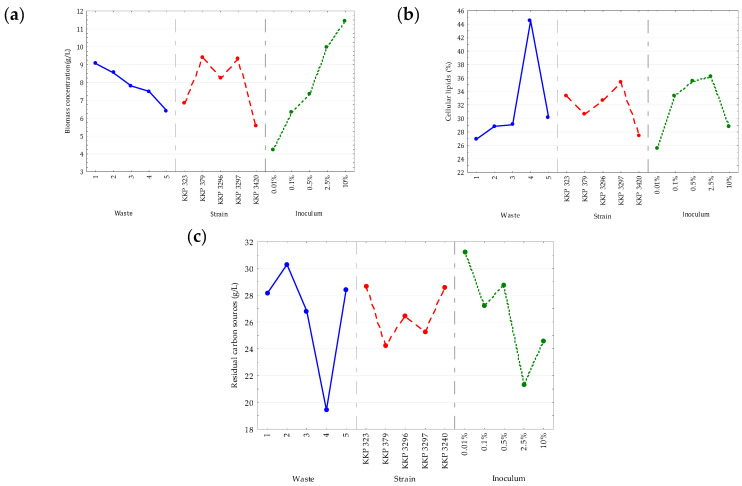
Main effects plot for the (**a**) biomass concentration (g/L), (**b**) cellular lipid content (%), and (**c**) residual carbon sources content in medium (g/L) when the cultures in mineral media varied in the types of wastes, *Y. lipolytica* strains, and sizes of the inoculum. 1—post-frying rapeseed oil from chicken frying, 2—waste lard from pork frying, 3—waste fat from pork head cooking, 4—solid lipid fraction from meat broths, and 5—post-frying rapeseed oil from chicken frying + waste lard (50:50). All of the culture variants were incubated at 28 °C for 96 h.

Generally, a lower biomass concentration was observed in all culture variants compared with the experiment in media with organic nitrogen sources. The statistical analysis indicated significant differences between the three studied variables and the obtained biomass concentration in the media. As shown in Figure 2a, the highest biomass yield was obtained in the culture medium with the addition of post-frying rapeseed oil from chicken frying (9.07 g/L). The result confirms observations made earlier. A slightly lower biomass concentration, but still above the average, was obtained when lard was used as a carbon source (8.54 g/L). Mixing post-frying oil with waste fat from pork head cooking in a ratio of 1:1 (m/m) resulted in the lowest biomass concentration of all tested variants (6.4 g/L). The more unsaturated fatty acids that were contained in the carbon source used, the more accessible it was to the enzymes. At this point, the fat was more fluid and easily digested [28]. The post-frying oil contained the most MUFAs (62%) and PUFAs (21%) (Table 1) and was the only waste that did not form aggregates in the culture medium.

It was also noted that a higher biomass concentration was significantly correlated with a higher inoculum volume (*p*-value = 0.0000) (Figure 2a). The highest volume of added inoculum (10%) resulted in a higher biomass concentration of 11.44 g/L. The indicated correlation was probably because adding more inoculum shortened the yeast’s lag phase (adaptive phase). Due to the higher number of active cells in the environment, the intensive division phase (log phase) was accelerated and the density of cells in the environment was increased [29]. The highest amount of biomass was generated by strains KKP 379 and KKP 3297, which amounted to 9.4 g/L and 9.3 g/L, respectively (*p*-value = 0.000).

The effect of the waste substrate in the culture medium was significant for the intracellular lipid content (*p*-value = 0.0022) (Figure 2b). The highest efficiency of the cellular lipid accumulation was correlated with the use of the solid lipid fraction from meat broths as a carbon source (Figure 2b), while at the same time, this waste was the most efficiently utilized by the yeast (Figure 2c). The *Y. lipolytica* strain and inoculum size did not significantly affect the variable studied. Thus, it can be said that in the medium with organic nitrogen sources (peptone and yeast extract), the efficiency of the cellular lipid accumulation was correlated with the initial concentration of the waste carbon source and the strain. On the other hand, in the mineral medium with inorganic nitrogen sources (ammonium sulfate), correlations were noted between the microbial lipid production efficiency and the type of waste carbon source.

Significant correlations were observed between the degree of lipid substrate utilization and the carbon source (*p*-value = 0.0010) and inoculum size (*p*-value = 0.0032) (Figure 2c. No differences were noted between the strains. The microorganisms most efficiently utilized the lipid solid fraction that remained after cooking. For this substrate, the least amount of unused lipid substrate was found in the medium, which accounted for 38.85% of the initial content. Higher inoculum doses (2.5% and 10%) were correlated with lower residual carbon sources in the medium. However, the correlation was not linear. The lipid substrates were used most efficiently at an inoculum size of 2.5%.

The described experiment showed that the accumulation of microbial lipids was closely related to the consumption of hydrophobic carbon sources in the culture medium. A greater consumption of available wastes resulted in a more efficient accumulation process. For the experiment conducted in media with a non-organic nitrogen source (Figure 2), the differences between the strains in the efficiency of microbial lipid accumulation were not significant. In contrast, the size of the inoculum significantly affected the growth and residue of the lipid carbon source in the medium. In Fabiszewska et al.’s [30] study, reducing the inoculum volume positively influenced the culture parameters in the media with hydrophobic carbon sources. In the olive oil medium, the cellular lipid content reached 34.1% when a lower inoculum volume (0.025% *v*/*v*) was used, which was nearly four times higher compared with the lipid content of 9% achieved with an inoculum volume of 0.075% *v*/*v*. An even greater difference was observed when waste fish oil served as the carbon source, with a sixfold increase in the lipid accumulation (8.9%) [30]. The results also indicate that the use of a smaller amount of inoculum positively affected the growth of the yeast cells, which contradicts the results obtained by Rakicka et al. [31]. The authors obtained a higher biomass yield using a lower-density inoculum. The researchers determined the possibility of lipid synthesis by the genetically modified yeast strain *Y. lipolytica* JMY4086 in media that contained molasses and waste glycerol. The experiments described by the authors showed that the lipid content in yeast cells was positively influenced by using an inoculum with a lower optical density and no oxygen regulation [31].

### 2.3. Evaluation of Extracellular Lipolytic Activity

**Figure 3:**
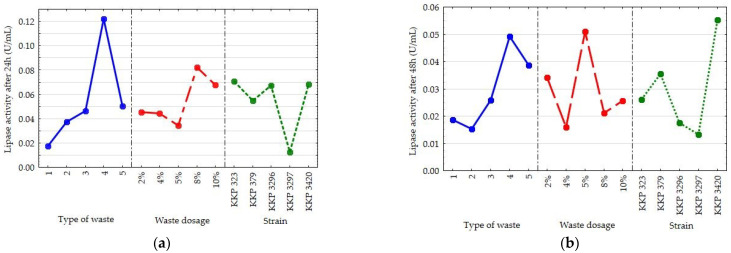
The main effects plots for the extracellular lipase activity (U/mL) (**a**) after 24 h, (**b**) after 48 h, when there were various types of lipid waste, *Y. lipolytica* strains, and sizes of inoculum applied.

Based on the first experimental design, the effects of the type of waste, its concentration, and the *Y. lipolytica* strain on the extracellular lipolytic activity of yeast cells after 24 and 48 h was additionally studied. After the first day of culture in the media with organic nitrogen sources (peptone and yeast extract), the factors that significantly affected the extracellular lipolytic activity were all the variables analyzed: carbon source, level of carbon source dosage, and type of strain (Figure 3a). A higher lipolytic activity correlated with the use of solid lipid carbon sources. The waste that specifically stimulated the lipase activity was the solid lipid fraction from the meat broths cooked after both 24 h and 48 h. The lipolytic activity of all the strains was higher on the first day of the experiment than after 48 h (Figure 3b). A higher extracellular lipolytic activity correlated with the use of a higher dosage of carbon source (8–10%).

The ability of all strains to hydrolyze hydrophobic substrates was compared. On the first day, the results for all strains were comparable, except for strain KKP 3297, which revealed the lowest ability to hydrolyze lipid substrates (Figure 3a). After 48 h, the lipolytic activity depended significantly on the types of carbon source and strain used (Figure 3b). It is noteworthy that the observed lipolytic activity in the case of the tested yeast strains significantly correlated with the biomass yield obtained, as well as the synthesized amount of intracellular lipids (Figure 1).

**Figure 4:**
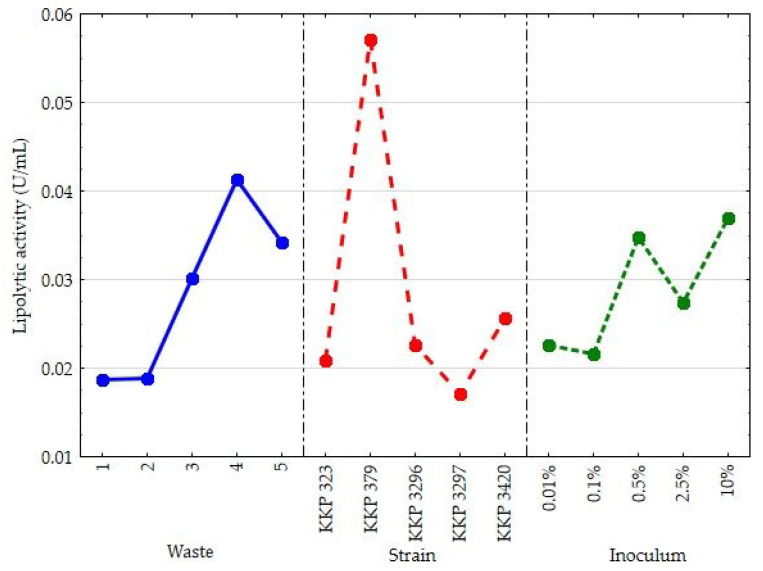
The main effects plot for extracellular lipase activity (U/mL) after 96 h of culture in media with peptone and yeast extract, when various types of lipid waste, *Y. lipolytica* strains, and sizes of inoculum were applied.

In the experiment based on the second experimental design, the effects of the type of waste, inoculum size, and *Y. lipolytica* strain on the extracellular lipolytic activity of yeast cells after 96 h of culture was also studied (Figure 4). The observed lipolytic activity of the yeast was not significantly dependent on the inoculum size. The calculated *p*-value indicates that significant statistical differences existed between the carbon source (*p*-value = 0.0122), the yeast strain *Y. lipolytica* (0.0000), and the analyzed activity. Once again, the solid lipid fraction from the meat broths was the best lipase inducer. Extraordinary lipolytic abilities were revealed for strain KKP 379 compared with the other yeast strains tested.

It is well known that microbial lipase production is strongly influenced by various culture conditions, including the presence of lipids, types of carbon and nitrogen sources, minerals, temperature, pH levels, inoculum, and activating or inhibitory compounds [32]. The experiments confirmed that lipid substrates and their amounts in the culture medium are crucial for high lipase yields. In a study by Lopes et al. [33], an increase in pH from 5.6 to 7.2 and an initial waste cooking oil concentration of 10 g/L provided the maximum extracellular lipase activity of *Y. lipolytica*. During the culture of *Y. lipolytica* yeast in media with glucose and WCO, the production of lipase increased until the end of the incubation [34]. In contrast, in media with stearin or olive oil, the lipase production reached a maximum value and decreased during the stationary phase of growth [35,36]. Although *Y. lipolytica* was able to produce lipase from stearin, an industrial derivative of tallow rich in saturated fatty acids, the production was still lower than in the presence of unsaturated fatty acids [35]. Moreover, a high proportion of lipid substrates in the medium may affect the oxygen transfer from the air to the culture and then inhibit the lipolytic activity [37].

Most authors consider olive oil an outstanding carbon source for stimulating the lipase synthesis of the yeast *Y. lipolytica*. The stimulating effect of olive oil on microbial lipase synthesis is attributed to its high oleic acid content [32]. However, it should be noted that extracellular lipases expressed by different strains of *Y. lipolytica* are not equally activated by the presence of vegetable oils [38]. Akpinar and Ucar [39] could not find simple correlations between the composition of the lipid substrate present in the medium and the hydrolytic activity during the culture of 22 different yeast strains of *Y. lipolytica* [39].

In the present study, solid lipid waste stimulated extracellular lipolytic activity better than liquid waste, despite its low unsaturated fatty acid content (Table 1). Lard after pork frying had the highest ratio of saturated to unsaturated fatty acids (SFAs/UFAs = 1). The solid lipid fraction after cooking was the waste that stimulated lipase production the most. Regarding the solid lipid substrates, it contained a high percentage of unsaturated fatty acids—60.47% (SFAs/UFAs = 0.65); only post-frying rapeseed oil from chicken frying contained more—83.21% (SFAs/UFAs = 0.20). Despite this high proportion of the unsaturated fraction, the post-frying rapeseed oil from chicken frying proved to be the least efficient waste in the production of lipases by *Y. lipolytica* yeast under the experimental conditions.

### 2.4. Emulsifying Abilities of Y. lipolytica Yeast in Waste Media

Based on the results of previous experiments, culture conditions with the best results in terms of a high biomass yield and efficient cellular lipid biosynthesis were chosen. Cultures were conducted in a mineral medium with 2.5% inoculum and 8% addition of waste carbon source. Two strains were selected: KKP 379 and KKP 323, as well as two wastes: the post-frying rapeseed oil from chicken frying was favorable considering the biomass increase, and the solid lipid fraction after cooking stimulated the efficient accumulation of cellular lipids.

The *Y. lipolytica* yeast efficiently degraded and utilized the substrates (meat industry waste) used in the culture media. Due to the consistency and composition of the lipid waste, the degree of medium homogeneity after 96 h of yeast culture varied. In the culture media that contained solid waste, aggregates of grease were visibly floating on the surface. The culture medium with post-frying oil was a milky emulsion with visible fat bubbles. In cultures with solid lipid fractions after cooking, partial emulsification of the substrate was also noticeable, in addition to visible aggregates of the lipid substrate. Granulometric analysis of the media after the cultures of strains KKP 379 and KKP 323 confirmed these observations. Only for the cultures with post-frying rapeseed oil from chicken frying and solid lipid fraction after cooking were it possible to assess the sizes of the fat particles distributed in the media.

**Figure 5:**
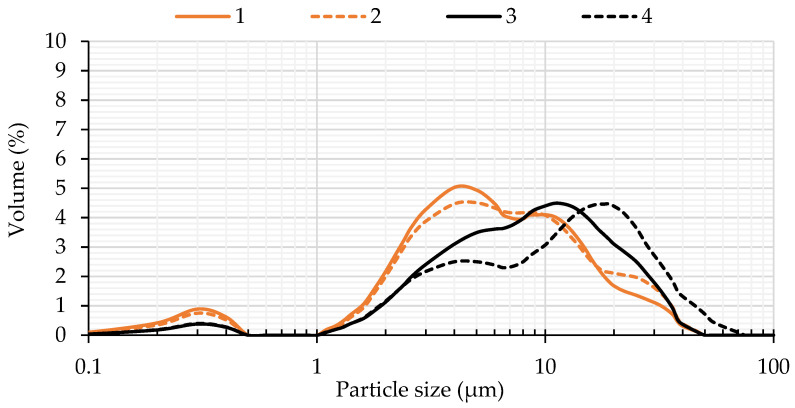
Particle size distribution (PSD) of post-frying rapeseed oil from chicken frying and solid lipid fraction after cooking in culture media after 96 h of incubation of *Y. lipolytica* KKP 379 and KKP 323 strains. 1—KKP 379 strain, post-frying rapeseed oil from chicken frying; 2—KKP 323, post-frying rapeseed oil from chicken frying; 3—KKP 379 strain, solid lipid fraction after cooking; 4—KKP 323, solid lipid fraction after cooking; 2.5%—size of inoculum; 8%—addition of carbon source.

Based on the dispersion factor (*span* = 2.51–3.23), no statistically significant differences were observed in the particle dispersions of the liquid and solid wastes used in the culture media (Table 2). However, for cultures in the media with solid lipid fractions after cooking, significantly higher values of *d*10, *d*50, and *d*90 were found. This means that the lipid particles in the media were larger than in the case of post-frying rapeseed oil from chicken frying (Figure 5). Comparing the *d*50 values for the cultures of the two strains, strain KKP 379 degraded the solid waste more efficiently than strain KKP 323. More lipid particles of a smaller size (*d*50 = 7.96 μm) were in the medium after the KKP 379 strain culture than in the medium with the same waste (*d*50 = 10.78 μm) for the KKP 323 strain. It is noteworthy that strain KKP 379 accumulated cellular lipids more efficiently, and the final biomass concentration was higher than for strain KKP 323 (Table 2). Similar observations were made for both medium variants, with liquid and solid wastes. In the media with post-frying rapeseed oil from chicken frying, differences in the particle size of the waste substrate after 96 h culture were not significant (*p*-value > 0.05). Incorporating emulsifiers, such as Tween 80, into the *Y. lipolytica* cultures is scientifically supported as a strategy to enhance lipase production, improve substrate utilization, and increase lipid accumulation. This approach leverages the yeast’s natural metabolic capabilities while optimizing the conditions for biotechnological applications [40].

Almost forty years ago, there were reports of emulsifier production by *C. lipolytica* ATCC 8662 in the presence of n-hexadecane. The emulsifier was active in an acid environment and stable up to 70 °C [41,42]. The tropical marine strain *Y. lipolytica* NCIM 3589 produced the emulsifier in the presence of alkanes. The complex was present in association with the cell wall and increased the hydrophobicity of the cell in the log phase. In contrast, extracellular synthesis of this emulsifier was observed in the stationary phase of ascension. However, the cell-associated and extracellular emulsifiers had similar properties. Subsequently, a number of emulsifiers have been reported from different *Yarrowia* strains, such as Yansan (IMUFRJ 50682) and Rufisan (UCP 0988) [43].

**Figure 6:**
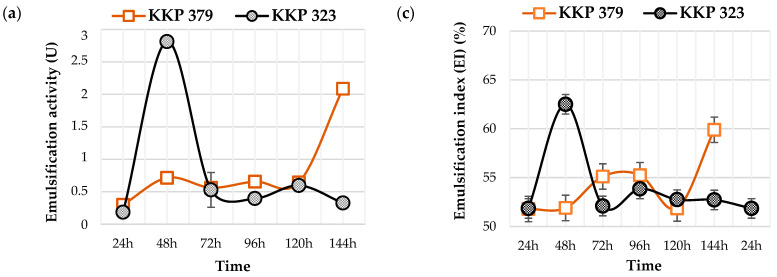

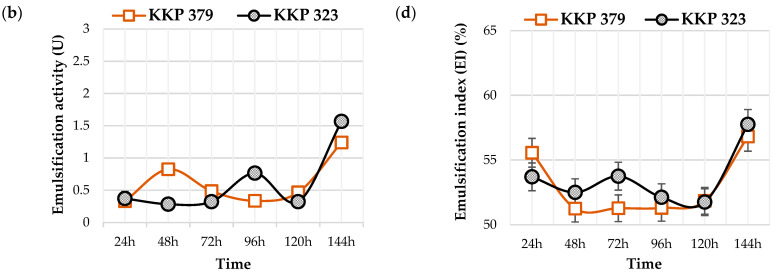
Emulsification activities and emulsification indexes for *Y. lipolytica* KKP 379 and KKP 323 strains during 144 h of cultivation in mineral medium with 8% addition of post-frying rapeseed oil from chicken frying (**a**,**b**) or solid lipid fraction after cooking (**c**,**d**).

The ability of *Y. lipolytica* yeast to form emulsions in media containing post-frying rapeseed oil from chicken frying or a solid lipid fraction after cooking as the primary carbon source is presented as an emulsification activity and emulsification index (Figure 6), which indicates the ability of a molecule to emulsify hydrocarbons [44].

The maximum emulsification activity was observed at the 48th hour of the KKP 323 culture in the medium with post-frying rapeseed oil from chicken frying. This corresponded to the cells entering the stationary phase of growth. It was also reflected in the value of the emulsification index (62%) (Figure 6c). When strain KKP 323 was cultured in the medium with solid waste fat, a slight increase in the emulsion activity was observed after 96 h of culture (Figure 6b). When solid waste was used as the carbon source, the emulsification activity was lower, which was also reflected in the lower emulsification index values (Figure 6d). Noteworthy for most variants, the increase in the emulsification activity was also noticeable after 144 h of yeast incubation. The maximum emulsifying activity of *C. lipolytica* ATCC 8662 in the medium with n-hexadecane was observed after 130 h of incubation [41]. Radha et al. [45] indicated that biosurfactant production by *Y. lipolytica* yeast is a secondary metabolic process. The best emulsifying activity (55%) was observed in the stationary phase of growth after 96 h of culturing the MTCC 9520 strain in a medium with chicken tallow as a low-cost carbon source. During the incubation of *Y. divulagata* NCAIM 1485 in a medium that contained 150 g/L glycerol, the emulsification index reached a maximum value of 69%. The index did not drop below 54% after 24 h, showing good stability [16].

To summarize, it is important to emphasize how important it is to use a given microorganism’s many properties in the targeted waste valorization. To obtain the desired oleaginous yeast metabolite in media with a lipid carbon source, not only the choice of waste, strain, and inoculum size but also the timing of the bioprocess and the composition of the culture medium, which were important factors that influenced the cell metabolism, proved to be crucial.

## 3. Materials and Methods

### 3.1. Chemicals

All reagents and solvents were obtained from Chempur (Piekary Śląskie, Poland). The fatty acid mixture standards used in the GC analysis were supplied by Sigma-Aldrich (Saint Louis, MO, USA). The PAH standards were acquired from Dr. Ehrenstorfer (Augsburg, Germany) and AccuStandard (New Haven, CT, USA).

### 3.2. Lipid Wastes Characterization

**Figure 7:**
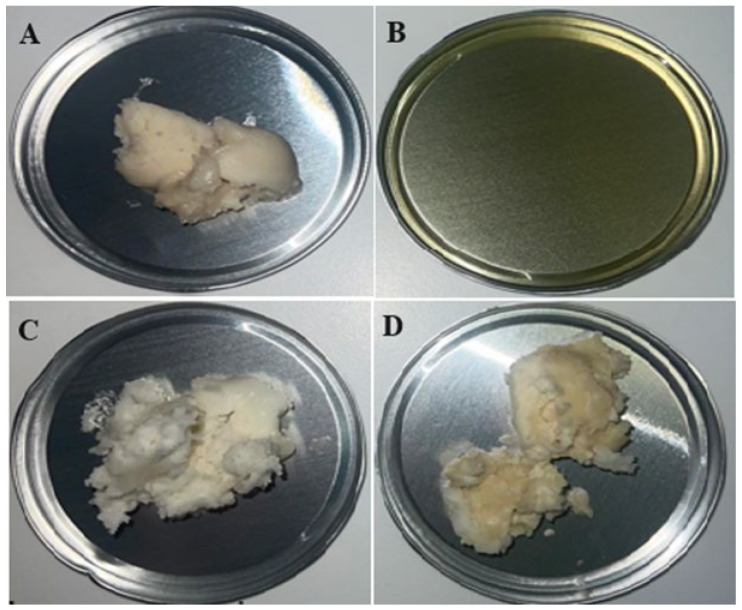
Waste from the meat industry: (**A**) solid lipid fraction from meat broths, (**B**) post-frying rapeseed oil from chicken frying, (**C**) waste fat from pork head cooking, and (**D**) waste lard from pork frying.

All the waste materials, namely, post-frying rapeseed oil from chicken frying, lard after pork frying, fat after cooking pork head, and solid lipid fraction after cooking, came from a meat-processing company in Świętokrzyskie Voivodeship (Poland) (Figure 7). The post-frying rapeseed oil from chicken frying came from fried poultry. The lard used in this study represents the residue from frying various pork parts. The solid lipid fraction after cooking was the residue from cooking bones, heads, pork tails, and fat collected from various broths: beef, pork, poultry, or mutton. To avoid releasing the fatty waste into the sewage system, it was collected separately. For the determination of the polycyclic aromatic hydrocarbons (PAHs) content in the selected wastes (Table 3), the method parameters described by Roszko et al. [46] were used. The fatty acid (FA) composition was analyzed by using a YL6100 GC gas chromatograph, as described by Wierzchowska et al. [20] according to the PN-EN ISO:2001 method (Table 1) [47].

### 3.3. Microorganisms and Culture Conditions

Five wild-type strains of *Yarrowia lipolytica*—KKP 323, KKP 379, KKP 3296, KKP 3297, and KKP 3420—were utilized in this study. The strains were obtained from the Collection of Industrial Microorganisms Cultures at the Professor Wacław Dąbrowski Institute of Agricultural and Food Biotechnology—State Research Institute in Warsaw, Poland. The yeast strains were preserved in a 20% (*v*/*v*) glycerol solution within YPG medium that contained 1% yeast extract, 2% peptone, and 2% glucose at −20 °C. Inoculation cultures were prepared in a YPG medium under controlled conditions. The cultures were incubated in 200 cm³ round-bottom flasks that contained 100 cm³ of medium at 28 °C for 24 h, with agitation at 140 rpm. To design the experiments, the Latin Square 5 × 5 method was employed as a statistical planning approach, where each experimental plan incorporated three variables at five different levels. Statistical analysis of the results was performed using Statistica 13.3 (Statsoft, Kraków, Poland).

### 3.4. Carbon Source Selection Experiment

The plan of this experiment involved three variables at five levels: yeast strain (KKP 323, KKP 379, KKP 3296, KKP 3297, KKP 3420), type of carbon source (post-frying rapeseed oil from chicken frying, waste lard from pork frying, waste fat from pork head cooking, solid lipid fraction from meat broths, post-frying rapeseed oil from chicken frying + waste lard (50:50)), and the level of the carbon source, as presented in Table 4. Each square in the table corresponds to a different variant of breeding, in which the levels of the three variables differed. All of the culture variants contained 20 g/L peptone and 10 g/L yeast extract. The 500 cm^3^ round-bottom flasks containing 200 cm^3^ of sterile medium were incubated at 28 °C for 96 h, with a rotation amplitude of 140 rpm. Statistical analysis of the results was performed using Statistica 13.3 (Statsoft, Poland).

### 3.5. Strain Selection Experiment

The plan of this experiment involved three variables at five levels: the yeast strain (KKP 323, KKP 379, KKP 3296, KKP 3297, KKP 3420), type of carbon source (post-frying rapeseed oil from chicken frying, waste lard from pork frying, waste fat from pork head cooking, solid lipid fraction from meat broths, post-frying rapeseed oil from chicken frying + waste lard (50:50)), and inoculum size (0.01%, 0.1%, 0.5%, 2.5%, 10%), as presented in Table 5. Each square in the table corresponds to a different variant of breeding, in which the levels of the three variables differed. All of the culture variants were incubated at 28 °C for 96 h with a rotation amplitude of 140 rpm in 500 cm^3^ round-bottom flasks that contained 200 cm^3^ of sterile mineral media that contained 1.5 g/L MgSO_4_, 0.16 g/L FeSO_4_ xH_2_O, 0.15 g/L CaCl_2_, 0.08 g/L MnCl_2_ x 4H_2_O, 0.02 g/L ZnSO_4_, 7 g/L KH_2_PO_4_, 3.5 g/L Na_2_HPO_4_, and 2.5 g/L (NH_4_)_2_SO_4_.

### 3.6. Analytical Techniques

The biomass concentration in the medium was determined based on the cell dry weight (CDW). The cells were harvested by centrifugation (8000 rpm (6869× *g*), 10 min) in an MPW-351R centrifuge (MPW Med. Instruments, Warsaw, Poland), washed with redistilled water, and dried at 80 °C. The dried samples of yeast biomass were crushed and homogenized in a laboratory grinder (Tube Mill control, IKA Poland Sp. z.o.o, Warsaw, Poland). Microbial lipids were extracted from the dry biomass using a modified Folch et al. [48] extraction method by treating the dried and washed biomass four times with portions of chloroform and methanol (2:1) and expressed in grams per gram of cell dry weight. Residue of hydrophobic substrates in the media after the culture was extracted by double straight extraction with portions of *n*-hexane. Next, the residual hydrophobic carbon source weight was measured after the evaporation of the solvents by distillation under a reduced pressure of 360 mbar (Buchi Rotavapor R-200 evaporator, Flawil, Switzerland). The carbon source residue was expressed in grams per liter of medium.

### 3.7. Lipase Activity Assay

The lipase activity was assessed using a modified spectrophotometric method, as previously described [30], based on the hydrolysis of p-nitrophenyl laurate. To evaluate the total activity of both the extracellular and cell-bound lipases, 1 mL of non-centrifuged culture was analyzed. Extracellular lipase activity was determined in supernatants (1 mL or 15 mL) obtained after centrifugation at 7000× *g* for 10 min. One unit of lipase activity was defined as the amount of enzyme required to release one µmol of p-nitrophenol per minute under the specified assay conditions.

### 3.8. Particle Size Distribution (PSD)

The droplet size distribution of hydrophobic substrates in culture media due to yeast activity was measured by laser diffraction using a CILAS 1190 particle size analyzer (CILAS, Orléans, France). The obscuration in all measurements was maintained at 5% using distilled water as a dispersant. The particle size distribution data included the particle diameter *d*50 and *span* calculations to determine the distribution width (Equation (1)):

(1) $$span=\frac{d90-d10}{d50}$$

where *d*90, *d*50, and *d*10 represent the mean particle size at the 90th, 50th, and 10th percentiles of the cumulative volume particle size distribution, respectively.

### 3.9. The Emulsification Activity Assay

The emulsification activity was assessed using a modified assay based on the method described by Cirigliano and Carman [41], with dodecane replacing hexadecane in the analysis. One unit of emulsification activity was defined as the quantity of emulsifier required to produce an emulsion with an absorbance of 1.0 at 540 nm under the specified conditions.

### 3.10. The Emulsification Index (EI)

The emulsification index (EI) was measured using a modified version of the method outlined by Cirigliano and Carman [41]. To determine the EI of the cell-free media samples, 1 mL of dodecane was mixed with an equal volume of the medium sample, followed by vortex-mixing for 2 min. The mixture was then allowed to settle for 10 min. The height of the emulsion layer that formed between the dodecane and aqueous phase was recorded. The EI was calculated as the percentage ratio of the emulsion layer height (cm) to the total liquid column height (cm).

## 4. Conclusions

For the growth of *Y. lipolytica* yeast, the most favorable was post-frying rapeseed oil from chicken frying, both in media with organic (peptone, yeast extract) and inorganic nitrogen (ammonium sulfate) sources. It can be said that for the efficient accumulation of cellular lipids, in media with inorganic nitrogen sources, there was a significant effect from the type of waste carbon source, while for culture in media with organic nitrogen sources, there were effects from the initial concentration of the carbon source and the strain. The solid lipid fraction from the meat broths was the waste that most stimulated the lipolytic activity of the *Y. lipolytica* strains and the efficient accumulation of intracellular lipids. The size of the inoculum did not significantly affect the efficiency of the lipid accumulation in the yeast cells. It had a significant effect on the growth and utilization rate of lipid substrates from the culture media. The differences in the substrate emulsification were evident in the media that contained different lipid solid wastes. Presumably, this could have been due to the varying ratio of saturated to unsaturated fraction. In the case of waste, such as lard, it is worth considering the addition of emulsifying agents to facilitate the breakdown of the substrate by yeast cells.

## Figures and Tables

**Table 1:** Contents of fatty acids in lipid wastes (%).

		Lipid Wastes			
	Fatty Acid	Post-Frying Rapeseed Oil from Chicken Frying	Lard After Pork Frying	Fat After Pork Head Cooking	Solid Lipid Fraction After Cooking
C10:0	Capric acid	n.d.	n.d.	0.27 ± 0.05 ^a^	0.04 ± 0.02 ^a^
C12:0	Lauric acid	n.d.	n.d.	0.17 ± 0.07 ^a^	0.09 ± 0.03 ^a^
C14:0	Myristic acid	0.76 ± 0.22 ^a^	2.14 ± 1.15 ^a^	2.06 ± 0.15 ^a^	0.49 ± 0.38 ^a^
C16:0	Palmitic acid	11.09 ± 0.32 ^a^	28.65 ± 6.87 ^b^	25.42 ± 0.44 ^b^	24.55 ± 1.00 ^b^
C16:1	Palmitooleic acid	0.71 ± 0.06 ^a^	1.90 ± 0.58 ^a^	1.97 ± 0.68 ^a^	3.57 ± 0.58 ^a^
C17:0	Margaric acid	n.d.	0.34 ± 0.06 ^a^	0.71 ± 0.18 ^a^	0.86 ± 0.58 ^a^
C17:1	Heptadecenoic acid	n.d.	0.24 ± 0.09	n.d.	n.d.
C18:0	Stearic acid	4.22 ± 0.10 ^a^	18.47 ± 1.01 ^b^	11.68 ± 1.03 ^b^	13.26 ± 1.05 ^b^
C18:1	Oleic acid	60.19 ± 0.95 ^b^	36.70 ± 5.59 ^a^	44.04 ± 0.52 ^a^	46.52 ± 2.35 ^a^
C18:2	Linoleic acid	16.21 ± 0.01 ^b^	9.25 ± 1.16 ^a^	4.38 ± 0.91 ^a^	5.56 ± 1.43 ^a^
C18:3	Linolenic acid	5.01 ± 0.36 ^b^	1.11 ± 0.43 ^a^	1.32 ± 0.08 ^a^	0.92 ± 0.06 ^a^
C20:0	Arachidic acid	0.73 ± 0.15 ^a^	0.40 ± 0.15 ^a^	5.25 ± 0.63 ^b^	0.14 ± 0.04 ^a^
C20:1	Eicosenoic acid	1.10 ± 0.17 ^a^	0.82 ± 0.42 ^a^	1.78 ± 0.05 ^a^	1.01 ± 0.11 ^a^
C20:3	Eicosatrienoic acid	n.d.	n.d.	0.21 ± 0.13 ^a^	0.06 ± 0.03 ^a^
C20:4	Arachidonic acid	n.d.	n.d.	0.48 ± 0.42	n.d.
C22:0	Behenic acid	n.d.	n.d.	0.17 ± 0.02 ^a^	0.10 ± 0.01 ^a^
C22:1	Erucic acid	n.d.	n.d.	0.08 ± 0.01 ^a^	0.14 ± 0.00 ^a^
SFAs	Saturated fatty acids	16.59 ± 0.80 ^a^	49.99 ± 6.92 ^b^	45.73 ± 0.76 ^b^	39.53 ± 0.21 ^b^
UFAs	Unsaturated fatty acids	83.21 ± 0.80 ^c^	50.01 ± 6.93 ^a^	54.27 ± 0.00 ^a^	60.47 ± 0.21 ^b^
MUFAs	Monounsaturated fatty acids	62.00 ± 1.18 ^b^	39.66 ± 5.33 ^a^	46.84 ± 0.22 ^ab^	50.93 ± 1.67 ^ab^
PUFAs	Polyunsaturated fatty acids	21.21 ± 0.38 ^b^	10.35 ± 1.59 ^a^	7.43 ± 1.12 ^a^	9.54 ± 1.46 ^a^
SFAs/UFAs		0.20	1.00	0.84	0.65

Different lowercase letters indicate significant differences between treatments (*p* < 0.05), n.d.—not detected

**Table 2:** Biomass concentration and microbial lipid accumulation efficiency in the cultures of *Y. lipolytica* KKP 379 and KKP 323 in media with post-frying rapeseed oil from chicken frying and solid lipid fraction after cooking and the volume particle size distribution (PSD) results.

No.	Strain	Carbon Source	*X* (g/L)	*Y* (%)	*d*10	*d*50	*d*90	*span*
1	KKP 379	Post-frying rapeseed oil from chicken frying	9.02 ± 0.64 ^b^	33.50 ± 1.50 ^b^	1.51 ± 0.13 ^a^	4.99 ± 0.55 ^a^	17.69 ± 6.68 ^a^	3.22 ± 0.96 ^a^
2	KKP 323		6.75 ± 0.35 ^ab^	24.20 ± 0.93 ^a^	1.79 ± 0.10 ^b^	5.62 ± 0.40 ^a^	20.17 ± 6.10 ^a^	3.23 ± 0.80 ^a^
3	KKP 379	Solid lipid fraction after cooking	8.57 ± 0.27 ^b^	34.66 ± 2.66 ^b^	2.32 ± 0.09 ^c^	7.96 ± 0.77 ^b^	23.23 ± 4.21 ^ab^	2.61 ± 0.28 ^a^
4	KKP 323		5.80 ± 0.61 ^a^	20.42 ± 1.64 ^a^	2.26 ± 0.10 ^c^	10.78 ± 1.04 ^c^	29.38 ± 3.49 ^c^	2.51 ± 0.10 ^a^

*X* (g/L)—biomass concentration; *Y* (%)—storage lipids yield per biomass; *d*90, *d*50, and *d*10 (μm)—particle size at the 90th, 50th, and 10th percentiles of the cumulative volume particle size distribution; 2.5%—size of the inoculum; 8%—addition of hydrophobic carbon source; lowercase letters indicate significant differences between the treatments (*p* < 0.05).

**Table 3:** Contents of polycyclic aromatic hydrocarbons (PAHs, μg/kg) in wastes used in yeast culture.

		Lipid Wastes			
	PAHs (µg/kg)	Post-Frying Rapeseed Oil from Chicken Frying	Lard After Pork Frying	Fat After Pork Head Cooking	Solid Lipid Fraction After Cooking
Naf	Naphtalene	4.43 ± 0.47	5.17 ± 0.52	5.15 ± 0.80	5.63 ± 0.20
Acy	Acenapthylene	0.26 ± 0.00	0.26 ± 0.01	0.33 ± 0.03	0.85 ± 0.04
Ace	Acenaphtene	0.67 ± 0.08	0.72 ± 0.08	0.61 ± 0.03	0.67 ± 0.11
Fluo	Fluorene	1.75 ± 0.24	1.44 ± 0.23	1.79 ± 0.32	1.65 ± 0.09
Phe	Phenanthrene	4.27 ± 0.68	4.34 ± 0.44	4.34 ± 0.48	4.98 ± 0.34
Ant	Anthracene	0.44 ± 0.03	0.30 ± 0.04	0.34 ± 0.03	0.54 ± 0.09
Car	Carbazole	0.47 ± 0.03	0.20 ± 0.00	0.17 ± 0.01	n.d.
Flua	Fluoranthene	0.83 ± 0.09	0.93 ± 0.18	0.92 ± 0.02	1.10 ± 0.06
Pyr	Pyrene	0.86 ± 0.03	0.91 ± 0.10	0.88 ± 0.02	1.29 ± 0.05
B[*a*]A	Benzo[*a*]anthracene	0.83 ± 0.12	0.41 ± 0.02	0.50 ± 0.03	0.35 ± 0.05
Chry	Chrysenes	0.84 ± 0.09	0.55 ± 0.05	0.53 ± 0.04	0.50 ± 0.05
B[*b*]F	Benzo[*b*]fluoranthene	0.38 ± 0.04	0.29 ± 0.04	0.30 ± 0.03	0.43 ± 0.03
B[*k*]F	Benzo[*k*]fluoranthene	1.22 ± 0.07	0.49 ± 0.04	0.78 ± 0.11	0.92 ± 0.09
B[*j*]F	Benzo[*j*]fluoranthene	0.86 ± 0.11	0.36 ± 0.04	0.63 ± 0.07	0.93 ± 0.08
B[*a*]P	Benzo[*a*]pyrene	0.72 ± 0.08	0.24 ± 0.03	0.45 ± 0.07	0.45 ± 0.04
IndP	Indeno [1,2,3-*cd*]pyrene	0.15 ± 0.01	0.26 ± 0.01	0.10 ± 0.01	0.09 ± 0.01
DB[*ah*]A	Dibenz[*a,h*]anthracene	0.16 ± 0.01	0.27 ± 0.03	0.11 ± 0.01	n.d.
DB[*ghi*]P	Benzo[*g,h,i*]pyrelene	0.24 ± 0.03	0.39 ± 0.06	0.04 ± 0.01	n.d.
**Σ**		19.38 ± 2.20 ^b^	17.53 ± 1.90 ^a^	17.98 ± 2.12 ^a^	20.39 ± 1.35 ^a^

Different lowercase letters indicate significant differences between treatments (*p* < 0.05). n.d.—not detected

**Table 4:** Design of cultures experiment in YP medium using a Latin Square plan 5 × 5 with three variables: the yeast strain, type of carbon source, and level of the carbon source.

Carbon Source	Yeast Strains					
	KKP 323	KKP 379	KKP 3296	KKP 3297	KKP 3420	
Post-frying rapeseed oil from chicken frying	20 g/L	40 g/L	50 g/L	80 g/L	100 g/L	Carbon source level
Waste lard from pork frying	40 g/L	50 g/L	80 g/L	100 g/L	20 g/L	
Waste fat from pork head cooking	50 g/L	80 g/L	100 g/L	20 g/L	40 g/L	
Solid lipid fraction from meat broths	80 g/L	100 g/L	20 g/L	40 g/L	50 g/L	
Post-frying rapeseed oil from chicken frying + waste lard (50:50)	100 g/L	20 g/L	40 g/L	50 g/L	80 g/L	

**Table 5:** Design of cultures experiment in mineral medium using Latin Square plan 5 × 5 with three variables: yeast strain, type of carbon source, and inoculum size.

Carbon Source	Yeast Strains					
	KKP 323	KKP 379	KKP 3296	KKP 3297	KKP 3420	
Post-frying rapeseed oil from chicken frying	0.01%	0.1%	0.5%	2.5%	10%	Carbon source level
Waste lard from pork frying	0.1%	0.5%	2.5%	10%	0.01%	
Waste fat from pork head cooking	0.5%	2.5%	10%	0.01%	0.1%	
Solid lipid fraction from meat broths	2.5%	10%	0.01%	0.1%	0.5%	
Post-frying rapeseed oil from chicken frying + waste lard (50:50)	10%	0.01%	0.1%	0.5%	2.5%	

## Data Availability

The original data presented in this paper are openly available in Wierzchowska, Katarzyna, 2024, “Biochemical pathways of storage lipid biosynthesis in oleogenic yeast cells by culture in hydrophobic carbon source media—a molecular insight”, RepOD, V1 at https://doi.org/10.18150/ZHKRNG.
